# The association between the weight-adjusted waist circumference index (WWI) and thyroid function: an NHANES study

**DOI:** 10.1007/s42000-026-00781-4

**Published:** 2026-04-23

**Authors:** Qiangbin Ding, Ke Li, Suqiong Lin, Rongliang Qiu, Guoyang Wu

**Affiliations:** 1 Department of General Surgery, Zhongshan Hospital of Xiamen University, Xiamen, China; 2 Department of Ultrasound Medical, Zhongshan Hospital of Xiamen University, Xiamen, China; 3 The School of Clinical Medicine, Fujian Medical University, Fuzhou, China

**Keywords:** NHANES, WWI, Thyroid function, Thyroid, Cross-sectional study, Obesity

## Abstract

**Introduction:**

Obesity has become a significant global public health issue that seriously threatens the health of more than one billion individuals worldwide. The weight-adjusted waist index (WWI) is a novel adiposity indicator calculated as waist circumference (cm) divided by the square root of body weight (kg). However, the association between WWI and thyroid function has not been reported to date. This study aims to investigate the relationship between WWI and thyroid function among U.S. adults.

**Methods:**

A total of 7,642 participants were enrolled in this study based on the National Health and Nutrition Examination Survey (NHANES) dataset from 2007 to 2012. Weighted statistical analyses were performed and three regression models were constructed sequentially, namely Model 1 (unadjusted), Model 2 (adjusted for age, sex, race, and survey cycle), and Model 3 (further adjusted for educational level, marital status, smoking status, alcohol consumption, diabetes mellitus, urinary iodine concentration, and poverty income ratio [PIR]). Stratified analyses and interaction effect tests were also conducted. Additionally, a retrospective validation cohort comprising 60 health check-up patients was extracted from Zhongshan Hospital Affiliated to Xiamen University between December 2024 and December 2025 to evaluate the association between WWI and free triiodothyronine (FT3) across WWI quartiles. The thyroid function indicators assessed included free triiodothyronine (FT3), free thyroxine (FT4), total triiodothyronine (TT3), total thyroxine (TT4), thyroid-stimulating hormone (TSH), thyroid peroxidase antibody (TPOAb), thyroglobulin antibody (TgAb), and thyroglobulin (Tg).

**Results:**

A total of 7,642 participants were included in this study. A positive correlation between WWI and FT3 levels was observed in Models 2 and 3, and this positive association persisted in both models after stratifying WWI into quartiles. The only exception was the WWI Q2 stratum in Model 3 where the 95% confidence interval (CI) for the association between WWI and FT3 included zero, indicating no statistical significance. Further stratified analyses revealed that the positive WWI-FT3 association was primarily evident in males, individuals under 50 years of age, and those of other Hispanic ethnicity. The positive WWI-FT3 correlation identified in NHANES was successfully replicated in our institutional validation cohort.

**Conclusion:**

A positive association between WWI and FT3 is suggested; however, further investigations are warranted to confirm this observation.

**Supplementary Information:**

The online version contains supplementary material available at https://doi.org/10.1007/s42000-026-00781-4.

## Background

 The global prevalence of overweight and obesity poses a significant public health challenge as excess adiposity is associated with increased risk of numerous chronic diseases [1, 2]. Traditional assessment relies on body mass index (BMI); however, BMI does not distinguish between fat and muscle mass, potentially misclassifying individuals with high muscle mass as overweight [3, 4]. To address this limitation, Park et al. proposed the weight-adjusted waist circumference index (WWI) in 2018 [5]. Unlike BMI, WWI reflects the fat-to-muscle ratio more accurately by adjusting waist circumference for body weight. Subsequent studies have demonstrated associations between elevated WWI and adverse health outcomes, including type 2 diabetes, cognitive impairment, depression, and cardiovascular disease in adults [6–10], as well as reduced bone mineral density in adolescents and adults [11, 12]. Nevertheless, the relationship between WWI and thyroid function is as yet a little explored area of study.

The thyroid gland represents a pivotal component of the endocrine system, responsible for secreting thyroid hormones that exert profound effects on metabolism, growth, and development, as well as the functioning of the nervous system [13, 14]. Research has shown that these hormones regulate cellular respiration, heat production, and basal metabolic rate, thereby influencing energy consumption. Additionally, obesity impacts thyroid function through various biological pathways, including by inducing lipotoxicity and altering the release of adipokines and inflammatory cytokines [15, 16]. Some studies indicate that obese individuals frequently exhibit elevated serum thyrotropin (TSH) concentrations, which may be related to increased infiltration of fat cells and lymphocytes within the thyroid gland associated with a rising BMI [17]. However, as mentioned above, investigation into the relationship between WWI and thyroid function has not to date been conducted to any great extent.

Few extensive epidemiological studies have explored the link between WWI and thyroid function in the general adult population. Using nationally representative U.S. data complemented by our institutional cohort data, this study aimed to delineate the relationship between WWI and thyroid function.

## Materials and methods

### The research population and design

**Fig. 1:**
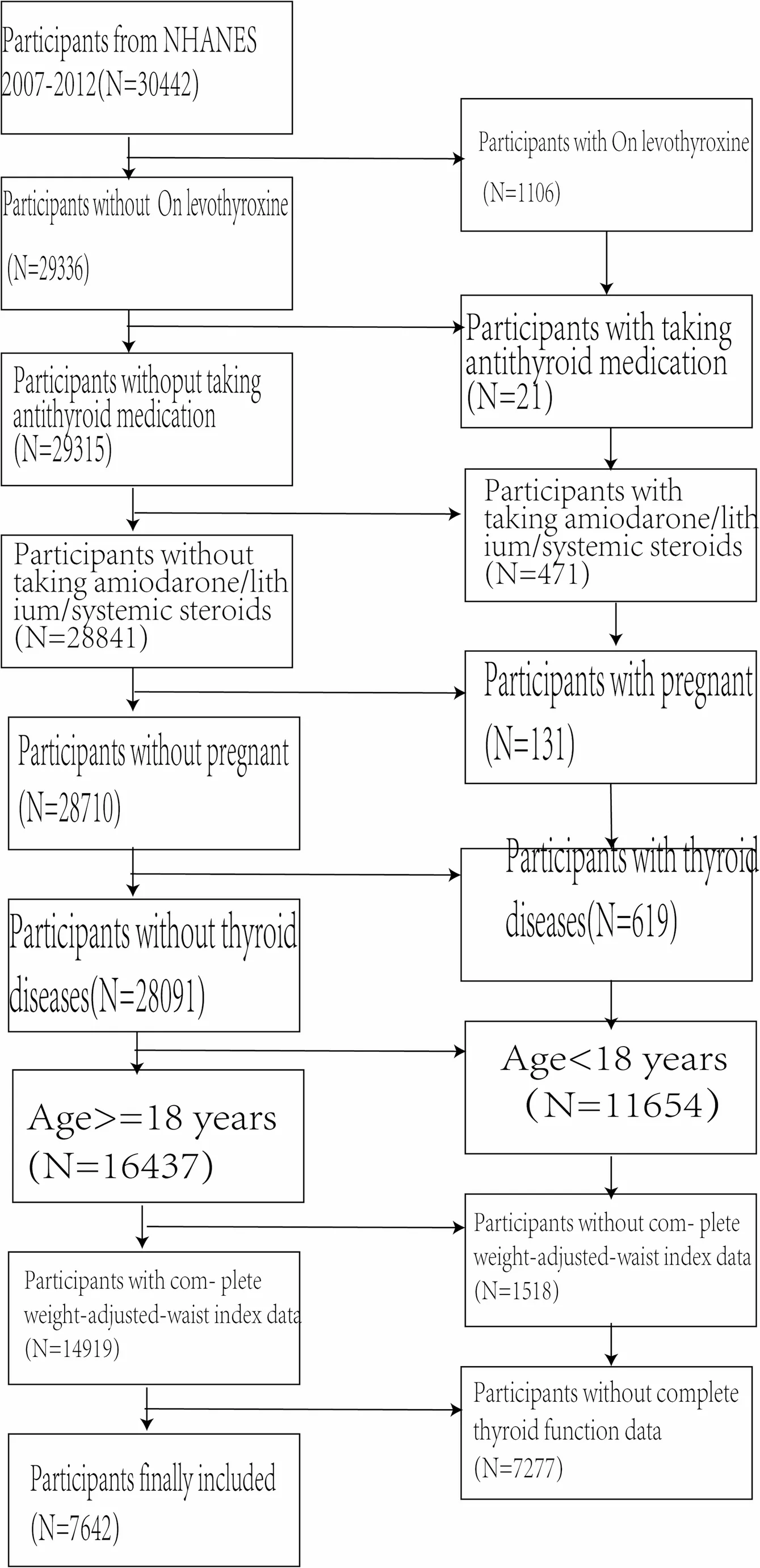
Participant selection flowchart NHANES = National Health and Nutrition Examination Survey

The National Health and Nutrition Examination Survey (NHANES) is a major survey program conducted by the Centers for Disease Control and Prevention (CDC) of the United States, designed to assess the health and nutritional status of American adults and children. The dataset is updated biennially. For more information about NHANES, please visit https://wwwn.cdc.gov/nchs/nhanes/default.aspx.This cross-sectional study utilized data from three survey cycles spanning 2007 to 2012. A total of 30,442 individuals were initially screened, of whom 7,642 participants were enrolled. The inclusion and exclusion criteria are presented in Fig. 1. Given its cross-sectional design, the “baseline” in this study refers only to the measurements obtained from participants at a single examination; it does not represent the starting point of follow-up nor does it imply a causal relationship.

### WWI measurement

WWI was computed as waist circumference (cm) divided by the square root of body weight (kg). Measurements were obtained by trained health technicians in the Mobile Examination Center (MEC). Participants were then stratified into quartile-based WWI groups (Q1–Q4).

### Thyroid function

Thyroid hormones (TSH, FT3, FT4, TT3, TT4, Tg, TPOAb, and TgAb) were quantified in accordance with the NHANES Laboratory/Medical Technologist Procedures Manual (LPM). All specimens were processed on a single immunoassay platform (Beckman Coulter Unicel DxI 800) and shipped to the University of Washington, Seattle, WA, USA. Reagent lots, calibrators, and quality-control parameters were updated every 2 years; cross-cycle calibration coefficients were supplied by the CDC through NHANES Bridging Guidance to ensure longitudinal comparability.

### Covariates

The covariates adjusted in the analyses were age, gender, race/ethnicity, survey cycle, educational attainment, marital status, smoking status, alcohol consumption, diabetes, urinary iodine concentration, and the poverty-to-income ratio (PIR). Diabetes status was ascertained from the NHANES medical conditions section, question identifier DIQ010.

### Institutional validation study

A total of 60 physical examination medical records from 2024 to 2025 in our hospital were retrospectively extracted. WWI was categorized into four groups and the independent-samples *t*-test and Cochran-Armitage trend test were performed to analyze the corresponding FT3 levels. This validation study was approved by the Hospital Ethics Committee of Zhongshan Hospital Affiliated to Xiamen University.

### Statistical analysis

All primary inferences of the current study were based on the association between free triiodothyronine (FT3) and WWI. Free thyroxine (FT4), total triiodothyronine (TT3), total thyroxine (TT4), thyroid-stimulating hormone (TSH), thyroid peroxidase antibody (TPOAb), thyroglobulin antibody (TgAb), and thyroglobulin (Tg) were treated as exploratory endpoints, with q-values reported following multiple comparison correction using the false discovery rate (FDR) method. All analyses were designated SDMVSTRA as the stratification variable and SDMVPSU as the primary sampling unit, with sampling weights examined using WTMEC2YR/3. All models were fitted using the svyglm function in the survey package to account for the impact of the complex sampling design on standard errors and confidence intervals, thus complying with the NHANES complex sampling framework. Statistical analyses were performed using EmpowerStats version 2.0 and R software version 4.2. Participants were categorized according to their WWI quartiles. Baseline characteristics were presented as mean ± standard deviation (SD). For laboratory indicators with skewed distributions (TSH, Tg, TPOAb and TgAb), median (interquartile range, IQR) were reported and weighted linear regression analyses were applied. Unadjusted and adjusted linear regression models were constructed, as follows: Model 1 was unadjusted for any covariates; Model 2 was adjusted for age, sex, race, and survey cycle; and Model 3 was further adjusted for age, sex, race, survey cycle, educational level, marital status, smoking status, alcohol consumption, diabetes mellitus, urinary iodine concentration, and the PIR. Restricted cubic spline (RCS) and generalized additive models (GAM) were used to fit the dose-response relationship between WWI and FT3, and non-linearity was assessed via the likelihood ratio test (LRT). A P-value < 0.05 in the LRT indicated the presence of non-linearity; otherwise, a linear relationship was observed. Segmented regression was performed as an exploratory threshold analysis. If the LRT P-value was > 0.05, the linear model was retained as the primary result. Stratified analyses were conducted to evaluate the differential effects of WWI on FT3 and interaction effects were assessed. Subgroup analyses by sex, age, and race were pre-specified during the study protocol design phase to evaluate effect modification. In each stratified model, the lowest WWI quartile (Q1) was set as the reference group within the subgroup. Statistical significance was defined as a two-sided P-value < 0.05.

## Results

### Baseline characteristics

After application of the pre-specified inclusion and exclusion criteria, 7,642 participants remained for analysis. Table 1 presents the weighted demographic and baseline characteristics across WWI quartiles. Except for alcohol intake and TPOAb, Tg, and TgAb levels, all variables differed significantly among WWI groups (*P* < 0.05). Compared with the lowest quartile, participants in the highest WWI quartile were older, more likely to be women, of Mexican-American or other Hispanic origin, less educated, married or living with a partner, and from lower-income households; they also exhibited a higher prevalence of diabetes and lower urinary iodine concentrations and were predominantly non-smokers. Laboratory analyses showed that individuals in the highest WWI quartile had significantly higher triglycerides, LDL-C, TT4, TSH, TgAb, and Tg levels than those in the lowest quartile (*P* < 0.05). Under the complex survey weights, the 7,642 included participants were, on average, older, more likely to be women, non-Hispanic White, slightly better educated, and wealthier, and had marginally lower BMI and smoking rates and higher urinary iodine concentrations than the 22,800 excluded individuals, whereas lipid and other metabolic indices were materially similar (Supplementary Table S6).

**Table 1:** Baseline characteristics of participants, weighted

WWI	Q1 *N* = 1911	Q2 *N* = 1910	Q3 *N* = 1910	Q4 *N* = 1911	*P*-value
Gender (%)					< 0.001
Male	59.296	55.133	53.295	40.116	
Female	40.704	44.867	46.705	59.884	
Age (years)	34.050 ± 13.140	43.712 ± 14.025	49.220 ± 15.124	56.269 ± 16.680	< 0.001
Race (%)					< 0.001
Mexican American	4.868	8.682	11.132	11.627	
Other Hispanic	4.273	6.065	6.512	6.637	
Non-Hispanic White	70.146	67.076	65.932	67.769	
Non-Hispanic Black	13.755	10.085	9.738	9.123	
Other Race - including Multi-Racial	6.959	8.092	6.686	4.843	
Educational level (%)					< 0.001
College graduate or above	31.961	40.052	48.247	55.887	
Less than college graduate	68.039	59.948	51.753	44.113	
Marital status (%)					< 0.001
Married/Living with partner	56.835	68.670	70.027	62.289	
Widowed/Separated	3.007	4.627	8.033	13.864	
Never married/Divorced	40.158	26.703	21.940	23.847	
PIR	3.093 ± 1.657	3.152 ± 1.670	2.939 ± 1.620	2.603 ± 1.634	< 0.001
BMI (kg/m^2)	24.272 ± 4.247	27.467 ± 5.009	30.112 ± 5.823	33.011 ± 7.357	< 0.001
Diabetes (%)					< 0.001
Yes	1.286	4.703	8.578	18.718	
Borderline	0.470	1.411	1.737	2.721	
No	98.244	93.885	89.685	78.561	
Previously diagnosed diabetes					< 0.001
HbA1c < 7%	79.2	74.4	68.9	61.2	
HbA1c ≥ 7%	20.8	25.6	31.1	38.8	
Smoking (%)					< 0.001
Now	46.462	42.896	35.961	34.814	
Some days	42.606	47.438	56.724	59.797	
Never	10.932	9.666	7.315	5.389	
Frequency of alcohol use in the last 12 months	3.906 ± 20.184	5.653 ± 28.715	7.100 ± 57.240	6.051 ± 49.518	0.158
Triglyceride (mg/dL)	104.180 ± 89.294	132.053 ± 112.337	150.315 ± 114.852	155.506 ± 112.109	< 0.001
LDL-cholesterol (mg/dL)	107.083 ± 33.589	116.660 ± 31.593	119.306 ± 34.541	120.256 ± 38.798	< 0.001
HDL-cholesterol (mg/dL)	56.343 ± 16.140	51.798 ± 16.158	48.923 ± 14.354	49.012 ± 14.192	< 0.001
FT3 (pg/mL)	3.261 ± 0.455	3.222 ± 0.569	3.203 ± 0.371	3.144 ± 0.524	< 0.001
FT4 (ng/dL)	0.795 ± 0.145	0.781 ± 0.130	0.782 ± 0.128	0.792 ± 0.165	0.003
TT3 (ng/dL)	115.882 ± 23.453	114.455 ± 23.669	115.597 ± 22.152	113.926 ± 25.483	0.038
TT4 (ug/dL)	7.586 ± 1.539	7.674 ± 1.456	7.830 ± 1.484	8.048 ± 1.636	< 0.001
TSH (mIU/L), IQR	1.39	1.45	1.56	1.7	< 0.001
TPOAb (IU/mL), IQR	0.60	0.60	0.60	0.60	1.000
TgAb (IU/mL), IQR	0.70	0.70	0.70	0.70	1.000
Tg (ng/mL), IQR	9.84	9.71	10.30	10.80	0.189
Urinary iodine concentration(UIC)(ug/mL)	152 (98–220)	148 (95–215)	145 (92–210)	140 (88–205)	0.042
cycle					
1	59.692	60.439	59.266	59.052	0.223
2	21.428	19.300	19.585	19.202	
3	18.881	20.261	21.149	21.746	

Table 1 has been supplemented with a WWI-quartile column. Group sizes and percentages are as follows: Q1 = 1 911 (25.01 %)（8.109–10.361.109.361）, Q2 = 1 910 (24.99 %)（10.361–10.952.361.952）, Q3 = 1 901 (24.99 %)（10.952–11.538.952.538）, Q4 = 1 911 (25.01 %)（11.538–14.096.538.096）, giving a total of 7,642 subjects. The WWI quartiles were generated with rankvar (WWI, 4), which splits the sample into four equally sized groups; cut-points were taken as the 25th, 50th, and 75th percentile values of the measured WWI, ensuring balanced group sizes.

Minimum-maximum ranges: FT3 3–28.84 pg/mL, FT4 0.9–4.8 ng/dL, TSH 0.276–69.836 mIU/L, TT3 121–632 ng/dL, TT4 8.8–27.6 µg/dL, TPOAb 0.283–2 376 IU/mL, Tg 0.353–4 461 ng/mL, and TgAb 0.7–1 895.4 IU/mL

All skewed indicators are presented as median (IQR), and normally distributed indicators as mean±SD.

### Association between WWI and the primary endpoint (FT3)

Table 2 presents the association results between WWI and FT3 across different models. In Model 1, which was unadjusted for any covariates, WWI was significantly negatively correlated with FT3, and this finding remained consistent after stratification by WWI quartiles. Model 2 showed a significant positive correlation between WWI and FT3, with the result still unchanged following quartile stratification. Further adjustment in Model 3 also demonstrated a positive association; however, after stratification by quartiles, this positive correlation was observed solely in the third and fourth quartiles, with no statistical significance detected in the second quartile.

**Table 2:** The association between WWI and FT3

	Model 1	*P*-value	Model 2	*P*-value	Model 3	*P*-value
	β(95% CI)		β(95% CI)		β(95% CI)	
FT3 (pg/mL)						
WWI	−0.054 (−0.067, −0.041)	< 0.001	0.060 (0.045, 0.075)	< 0.001	0.069 (0.045, 0.094)	< 0.001
WWI (per 1-SD increase)	−0.046 (−0.057, −0.035)	< 0.001	0.051 (0.038, 0.064)	< 0.001	0.059 (0.038, 0.080)	< 0.001
WWI (per IQR increase)	−0.064 (−0.079, −0.048)	< 0.001	0.071 (0.053, 0.088)	< 0.001	0.081 (0.053, 0.111)	< 0.001
Categories						
Q1	0		0		0	
Q2	−0.039 (−0.068, −0.011)	0.007	0.055 (0.027, 0.083)	< 0.001	0.014 (−0.032, 0.060)	0.556
Q3	−0.059 (−0.089, −0.029)	< 0.001	0.089 (0.058, 0.119)	< 0.001	0.091 (0.043, 0.139)	<0.001
Q4	−0.117 (−0.149, −0.086)	< 0.001	0.121 (0.087, 0.156)	< 0.001	0.126 (0.071, 0.182)	< 0.001
***p*** for trend		< 0.001		< 0.001		< 0.001

Model 1: No covariates were adjusted

Model 2: Adjusted for: age, gender, race, and cycle

Model 3: Adjusted for: age, gender, race, cycle, education level, marital status, alcohol consumption, diabetes, smoking, UIC, and PIR

β = change in outcome units per 1-unit increase in WWI

To explore potential threshold effects, we performed an exploratory piecewise linear regression, with the inflection point *K* estimated by the maximum likelihood method. As shown in Supplementary Tables 8 and 9, as well as in Supplementary Fig. 2, although both Model 2 and Model 3 estimated the inflection point *K* to be 11.687, the likelihood ratio test (LRT) indicated no statistically significant difference between the two-segment model and the single linear model (*P* > 0.05). Therefore, the continuous linear model was retained as the basis for primary inference and it was concluded that there was no significant threshold effect between WWI and FT3.

### Association of WWI with exploratory thyroid indicators

As an exploratory analysis, we evaluated the relationships between WWI and other thyroid indicators (Supplementary Table S3). After FDR correction, WWI remained significantly and positively associated with TT3, TT4, and Ln-Tg (q < 0.05), whereas associations with FT4, TSH, TPOAb, and TgAb did not survive multiple testing correction. These findings are considered preliminary and require validation in an independent cohort. Missing data imputation (MI) results are presented in Supplementary Table S7. Details regarding the sign reversal across different levels of adjustment are provided in Supplementary Table S1. To assess the impact of residual confounding on the WWI-FT3 association, we performed sensitivity analyses. The direction of the association between WWI and FT3 in both sensitivity analysis populations was consistent with that in the main analysis (Supplementary Tables S4 and S5).

### Stratified analyses

To assess the stability of the relationship between WWI and FT3, we conducted in-depth stratified analyses (Table 3). The overall positive association between WWI and FT3 exhibited significant heterogeneity across sex and race subgroups and was also pronounced in individuals aged < 50 years; no significant differences were observed in other stratified analyses by educational level, marital status, diabetes status, or smoking status.

**Table 3:** Effect size of WWI on FT3 in pre-specified and exploratory subgroups

Character	*N* (7,642)	WWI		Categories WWI				
		95% CI	*P* for interaction	Q1	Q2	Q3	Q4	*P* for interaction
Gender			0.713					0.002
Male	**4093**	0.074 (0.039, 0.108)		Reference	0.050 (−0.008, 0.108)	0.119 (0.056, 0.182)	0.088 (0.010, 0.166)	
Female	**3549**	0.083 (0.048, 0.118)		Reference	−0.518 (−0.782, −0.255)	−0.419 (−0.683, −0.155)	−0.302 (−0.563, −0.041)	
Age (years)			0.193					0.019
< 50	**4238**	0.050 (0.018, 0.081)		Reference	−0.030 (−0.084, 0.025)	0.050 (−0.010, 0.110)	0.144 (0.062, 0.225)	
>=50	**3404**	0.017 (−0.021, 0.055)		Reference	−0.211 (−0.426, 0.005)	−0.183 (−0.397, 0.031)	−0.195 (−0.403, 0.013)	
Race			< 0.001					< 0.001
Mexican American	**1295**	0.026 (−0.073, 0.125)		Reference	−0.028 (−0.234, 0.179)	0.051 (−0.153, 0.255)	−0.028 (−0.259, 0.204)	
Other Hispanic	**883**	0.370 (0.243, 0.497)		Reference	−0.023 (−0.720, 0.674)	0.169 (−0.542, 0.881)	0.802 (0.078, 1.526)	
Non-Hispanic White	**3332**	0.060 (0.032, 0.089)		Reference	−0.063 (−0.524, 0.399)	0.009 (−0.453, 0.471)	0.015 (−0.447, 0.477)	
Non-Hispanic Black	**1577**	0.060 (−0.011, 0.131)		Reference	−0.305 (−0.946, 0.336)	−0.186 (−0.833, 0.460)	−0.125 (−0.775, 0.526)	
Others	**555**	0.066 (−0.053, 0.184)		Reference	−0.137 (−0.841, 0.566)	−0.033 (−0.800, 0.735)	−0.017 (−0.796, 0.763)	
Educational level			0.538					0.642
Less than college graduate	**6159**	0.073 (0.047, 0.100)		Reference	0.018 (−0.034, 0.070)	0.099 (0.044, 0.153)	0.144 (0.083, 0.205)	
College graduate or above	**1483**	0.057 (0.008, 0.106)		Reference	0.044 (−0.309, 0.396)	0.103 (−0.251, 0.457)	0.080 (−0.275, 0.436)	
Marital status			0.616					0.145
Married/Living with partner	**4412**	0.064 (0.033, 0.096)		Reference	0.054 (−0.005, 0.112)	0.090 (0.029, 0.150)	0.140 (0.069, 0.212)	
Widowed/Separated	**759**	0.041 (−0.051, 0.132)		Reference	−0.096 (−0.532, 0.341)	0.043 (−0.381, 0.467)	−0.003 (−0.446, 0.440)	
Never married/Divorced	**2076**	0.084 (0.041, 0.128)		Reference	−0.104 (−0.390, 0.181)	0.069 (−0.219, 0.357)	0.071 (−0.214, 0.356)	
Diabetes			0.756					0.946
Borderline	**126**	0.020 (−0.269, 0.310)		Reference	0.135 (−0.529, 0.799)	0.256 (−0.295, 0.808)	0.144 (−0.460, 0.748)	
Yes	**818**	0.038 (−0.053, 0.129)		Reference	−0.243 (−1.331, 0.845)	−0.243 (−1.327, 0.841)	−0.169 (−1.258, 0.920)	
No	**6698**	0.070 (0.044, 0.097)		Reference	−0.138 (−1.128, 0.851)	−0.059 (−1.049, 0.931)	−0.026 (−1.017, 0.964)	
Smoking			0.179					0.184
Now	**1345**	0.057 (0.020, 0.094)		Reference	0.023 (−0.048, 0.094)	0.132 (0.055, 0.208)	0.082 (−0.005, 0.168)	
Some days	**280**	0.007 (−0.079, 0.093)		Reference	0.032 (−0.504, 0.568)	0.089 (−0.469, 0.648)	0.194 (−0.373, 0.762)	
Never	**6017**	0.087 (0.051, 0.123)		Reference	−0.051 (−0.321, 0.219)	0.024 (−0.247, 0.294)	0.097 (−0.176, 0.369)	

β represents change in FT3 (pg/mL) per 1-unit increase in WWI

Q1 (lowest WWI quartile) served as the reference group (Ref) within each stratum

### Institutional validation study

**Fig. 2:**
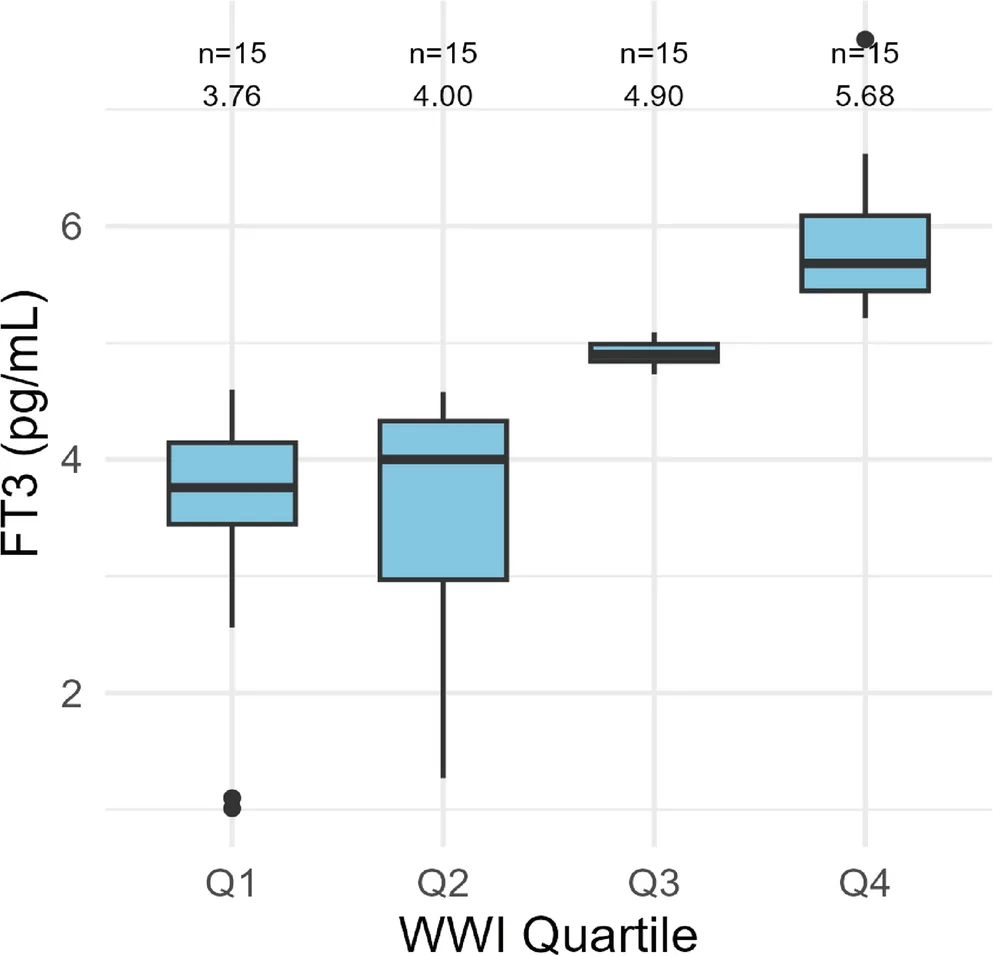
External validation: boxplot of WWI quartiles vs. FT3 levels (n = 60)

Medical records of health check-ups conducted at our hospital between 2024 and 2025 were retrospectively retrieved. Quartile boxplots (Fig. 2) demonstrated that the directional association between WWI and FT3 was consistent with the findings from the NHANES dataset.

## Discussion

To the best of our knowledge, this is the first study to investigate the association between WWI and thyroid function using a nationally representative sample of U.S. adults. Furthermore, based on 60 cases from our hospital, this study is the first to validate this association simultaneously in a large-scale population and an independent clinical cohort. Among the 7,642 participants, Model 2 demonstrated a positive correlation between WWI and FT3, which persisted after stratification by WWI quartiles. Further adjustment in Model 3 also showed a positive association; however, after quartile stratification, this positive correlation was observed only in the third and fourth quartiles. After the stringent exclusion of participants using medications, with diseases, or with abnormal thyroid function, the WWI-FT3 association remained a stable positive dose-response relationship. This relationship was also confirmed in the 60 cases from our hospital. Stratified analyses indicated significant heterogeneity in the overall positive association between WWI and FT3 across sex and race subgroups, and this association was also pronounced in individuals aged < 50 years. Notably, in the WWI Q2 stratum of Model 3, the 95% confidence interval (CI) for the WWI-FT3 association included zero, indicating no statistical significance. It is important to note that although an exploratory analysis estimated a potential inflection point (K = 11.687), this was not supported by statistical testing (*P* > 0.05). Therefore, 11.687 should not be interpreted as a true biological threshold.

The association between obesity and thyroid function represents a well-established bidirectional relationship in medical research [18]. Research conducted by Radetti and colleagues in a cross-sectional study found that obese children frequently have thyroid structure and function irregularities that are not entirely attributable to autoimmune factors [19]. Studies also reveal that obese Chinese adults, even with normal thyroid function levels, exhibit elevated FT3 levels and a higher FT3/FT4 ratio compared to their non-obese counterparts [20]. Conversely, a Boston-based study observed that although serum TSH levels are generally higher in obese individuals compared to lean controls, these levels do not seem to be a mutable risk factor for metabolic syndrome among the obese and overweight population [21]. Hence, there is significant value in conducting extensive nationwide research to verify the potential link between obesity and thyroid function.

The relationship between obesity and thyroid function is extremely complex and involves numerous physiological and metabolic pathways [22]. The hypothalamic-pituitary-thyroid (HPT) axis is mainly responsible for regulating the production and release of thyroid hormones to maintain normal metabolism and development [23, 24]. If there is excessive secretion of thyroid hormones, it will enhance the body’s ability to break down fat and blood sugar, accelerate the metabolic process of these substances, and lead to unintentional weight loss [25, 26]. Conversely, when there is insufficient secretion of thyroid hormones, it will slow down the metabolic rate of fat and blood sugar, causing fat and blood sugar to accumulate, leading to obesity [27]. The subtypes of thyroid hormone receptors mainly include α, β, and β2 [28–30]. Among them, TRβ can control some genes and enzymes required for the proliferation of preadipocytes and the differentiation of adipocytes through peroxisome proliferator-activated receptor γ (PPAR γ), and thereby regulate the formation of fat [31–33]. Deiodinase D2 (type 2 iodothyronine deiodinase) is a selenium-containing enzyme. It can affect lipid accumulation in adipose tissue by catalyzing the conversion of T4 to T3 and further act on the transcription factor CCAAT/enhancer-binding protein (C/EBPs) [34–36]. In addition, leptin is a hormone-like protein secreted by adipocytes and can directly or indirectly affect thyroid function [37, 38]. Following a direct pathway, leptin acts on thyrotropin-releasing hormone (TRH) neurons expressing ObRb through its receptor ObRb, activates the phosphorylation of transcription factor signal transducer and activator of transcription 3 (STAT3) through the P - STAT3 signaling pathway, and promotes the binding of STAT3 to the promoter region of the TRH encoding gene, thereby increasing the biosynthesis and release of TRH. Following an indirect pathway, leptin acts on the arcuate nucleus to activate the melanocortin pathway and stimulate the release of TRH from TRH neurons located in the paraventricular nucleus [39, 40].

The current study utilized data from the rigorously designed and quality-controlled NHANES cross-sectional database, which employs multistage probability sampling and is nationally representative. Because serum insulin was not measured in NHANES 2007–2012, insulin resistance could not be directly assessed; we therefore used physician-diagnosed diabetes as a surrogate. The cross-sectional design precludes causal inference; thus, reverse causation as well as unmeasured confounding may persist. Selection, recall, and reporting bias cannot be excluded. Additionally, our institutional validation comprised only 60 retrospectively identified cases, limiting statistical power. Prospective studies with larger samples are warranted to confirm these findings. FT3 was designated as the primary endpoint, with all other thyroid biomarkers treated as exploratory outcomes. The FDR method was employed to control the error rate of multiple comparisons. Consequently, any significant associations identified beyond FT3 should be interpreted as suggestive and should not be regarded as confirmatory evidence.

## Conclusion

Our findings indicate a positive association between WWI and FT3; however, prospective epidemiological studies are required to confirm this observation.

## Supplementary Information

Below is the link to the electronic supplementary material.

Supplementary Material 1 (554 KB)

Supplementary Material 2 (897 KB)

Supplementary Material 3 (DOCX 40.5 KB)

## Data Availability

The datasets generated and/or analyzed during the current study are available in the NHANES repository, https://wwwn.cdc.gov/nchs/nhanes/default.aspx.
