# Supplementary material for: The association between the weight-adjusted waist circumference index (WWI) and thyroid function: an NHANES study
Source: Hormones (Athens). 2026 Apr 23;25(2):505–14. doi: 10.1007/s42000-026-00781-4 (PMC13582219; doi:10.1007/s42000-026-00781-4)
Supplement: Supplementary file 1 [file 42000_2026_781_MOESM3_ESM.docx]

Table S1. WWI-FT3 association with extended covariates (Model 4) – exploratory mechanistic check

|  | **Model 1** | ***P*-value** | **Model 4** | ***P*-value** |
| --- | --- | --- | --- | --- |
|  | **β(95% CI)** |  | **β(95% CI)** |  |
| **FT3 (pg/mL)** |  | | | |
| **WWI** | -0.054 (-0.067, -0.041) | <0.00001 | 0.090 (0.036, 0.145) | 0.00122 |
| **categories** |  |  |  |  |
| **Q1** | 0 | | 0 | |
| **Q2** | -0.039 (-0.068, -0.011) | 0.00740 | 0.016 (-0.068, 0.100) | 0.70374 |
| **Q3** | -0.059 (-0.089, -0.029) | 0.00013 | 0.107 (0.011, 0.203) | 0.02870 |
| **Q4** | -0.117 (-0.149, -0.086) | <0.00001 | 0.208 (0.090, 0.325) | 0.00055 |
| ***p* for trend** |  | <0.00001 |  | 0.00028 |

Model 4: adjust for: age, gender, race, cycle,Education level,marital status,alcohol consumption,diabetes,smoking,and the PIR,BMI,LDL-C，HDL-C,Triglycerides

This model is used solely to verify the sign reversal across different adjustment levels

Table S2. VIF and Pearson correlation matrix

|  | VIF | WWI | BMI | TG | LDL-C | HDL-C |
| --- | --- | --- | --- | --- | --- | --- |
| WWI | 1.39 | 1.00 | 0.488 | 0.195 | 0.144 | -0.179 |
| BMI | 1.39 | 0.488 | 1.00 | 0.156 | 0.090 | -0.292 |
| TG | 1.34 | 0.195 | 0.156 | 1.00 | 0.189 | -0.391 |
| LDL-C | 1.05 | 0.144 | 0.090 | 0.189 | 1.00 | -0.028 |
| HDL-C | 1.29 | -0.179 | -0.292 | -0.391 | -0.028 | 1.00 |

Table S3. Exploratory associations between WWI and other thyroid parameters.

|  | **Model 1** | ***P*-value** | **FDR-q** | **Model 2** | ***P*-value** | **FDR-q** | **Model 3** | ***P*-value** | **FDR-q** |
| --- | --- | --- | --- | --- | --- | --- | --- | --- | --- |
|  | **β(95% CI)** |  |  | **β(95% CI)** |  |  | **β(95% CI)** |  |  |
| **FT4 (ng/dL)** | -0.003 (-0.007, 0.001) | 0.12390 | 0.372 | -0.005 (-0.010, -0.001) | 0.02338 | 0.069 | -0.004 (-0.013, 0.004) | 0.29806 | 0.894 |
| **TT3 (ng/dL)** | -0.830 (-1.480, -0.181) | 0.01226 | 0.036 | 3.982 (3.232, 4.732) | <0.00001 | 0.001 | 4.070 (2.737, 5.404) | <0.00001 | 0.001 |
| **TT4 (ug/dL)** | 0.208 (0.166, 0.250) | <0.00001 | 0.001 | 0.219 (0.169, 0.269) | <0.00001 | 0.001 | 0.148 (0.059, 0.236) | 0.00106 | 0.003 |
| **TSH (mIU/L)** | 0.149 (0.105, 0.193 | <0.00001 | 0.001 | 0.047 (-0.006, 0.100) | 0.08407 | 0.092 | 0.052 (-0.037, 0.141) | 0.25495 | 0.936 |
| **LnTSH** | 0.074 (0.056, 0.091) | <0.00001 | 0.001 | 0.023 (0.003, 0.044) | 0.02412 | 0.069 | 0.043 (0.007, 0.080) | 0.02060 | 0.063 |
| **TPOAb(IU/mL)** | 0.886 (-1.347, 3.119) | 0.43678 | 0.481 | -1.775 (-4.461, 0.912) | 0.19552 | 0.216 | -5.748 (-10.814, -0.682) | 0.02622 | 0.078 |
| **LnTPOAb** | 0.010 (-0.035, 0.054) | 0.66686 | 0.734 | -0.075 (-0.128, -0.021) | 0.00612 | 0.018 | -0.111 (-0.203, -0.020) | 0.01719 | 0.051 |
| **TGAb(IU/mL)** | 2.240 (0.485, 3.995) | 0.01240 | 0.036 | 0.951 (-1.164, 3.066) | 0.37837 | 0.416 | -1.442 (-4.979, 2.095) | 0.42433 | 0.894 |
| **LnTGAb** | 0.043 (0.017, 0.068) | 0.00094 | 0.003 | -0.013 (-0.043, 0.017) | 0.39862 | 0.439 | -0.019 (-0.068, 0.031) | 0.45475 | 0.894 |
| **Tg (ng/mL)** | 2.873 (1.921, 3.825) | <0.00001 | 0.001 | 2.205 (1.062, 3.349) | 0.00016 | 0.001 | 0.671 (-1.007, 2.350) | 0.43311 | 0.894 |
| **LnTg** | 0.055 (0.029, 0.081) | 0.00005 | 0.001 | 0.082 (0.051, 0.113) | <0.00001 | 0.001 | 0.064 (0.014, 0.115) | 0.01201 | 0.036 |

Model 1: no covariates were adjusted.

Model 2: adjust for:age, gender, race, cycle.

Model 3: adjust for: age, gender, race, cycle,Education level,marital status,alcohol consumption,diabetes,smoking,and the PIR.

β= the change in outcome units per 1-unit increase in WWI.

Benjamini–Hochberg FDR correction was applied within each model column; q < 0.05 was considered robust.

Table S4. Association between WWI and FT3 in the normal-thyroid subset (n = 170)

|  | **Model 1** | ***P*-value** | **Model 2** | ***P*-value** | **Model 3** | ***P*-value** |
| --- | --- | --- | --- | --- | --- | --- |
|  | **β(95% CI)** |  | **β(95% CI)** |  | **β(95% CI)** |  |
| **FT3 (pg/mL)** |  | | | |  |  |
| **WWI** | 0.099 (0.021, 0.177) | 0.01377 | 0.080 (0.000, 0.160) | 0.05083 | 0.059 (0.010, 0.108) | 0.024 |
| **categories** |  |  |  |  |  |  |
| **Q1** | 0 | | 0 | | 0 | |
| **Q2** | 0.070 (-0.096, 0.237) | 0.40854 | 0.074 (-0.089, 0.237) | 0.37267 | 0.058 (0.009, 0.107) | 0.024 |
| **Q3** | 0.117 (-0.049, 0.284) | 0.16936 | 0.106 (-0.059, 0.271) | 0.21042 | 0.071 (0.018, 0.124) | 0.012 |
| **Q4** | 0.185 (0.019, 0.351) | 0.02990 | 0.149 (-0.018, 0.317) | 0.08236 | 0.065 (0.013, 0.117) | 0.018 |
| ***p* for trend** |  | 0.01377 |  | 0.05083 |  | 0.021 |

Table S5.Association between WWI and FT3 in the drug-clean population (n = 16 437)

|  | **Model 1** | ***P*-value** | **Model 2** | ***P*-value** | **Model 3** | ***P*-value** |
| --- | --- | --- | --- | --- | --- | --- |
|  | **β(95% CI)** |  | **β(95% CI)** |  | **β(95% CI)** |  |
| **FT3 (pg/mL)** |  | | | |  |  |
| **WWI** | -0.055 (-0.070, -0.040) | <0.00001 | 0.061 (0.045, 0.078) | <0.00001 | 0.066 (0.039, 0.092) | <0.00001 |
| **categories** |  |  |  |  |  |  |
| **Q1** | 0 | | 0 | | 0 | |
| **Q2** | -0.051 (-0.080, -0.022) | 0.00056 | 0.045 (0.016, 0.073) | 0.00193 | 0.011 (-0.035, 0.057) | 0.65187 |
| **Q3** | -0.055 (-0.085, -0.026) | 0.00028 | 0.089 (0.058, 0.119) | <0.00001 | 0.077 (0.028, 0.125) | 0.00198 |
| **Q4** | -0.118 (-0.150, -0.087) | <0.00001 | 0.118 (0.084, 0.152) | <0.00001 | 0.122 (0.067, 0.177) | 0.00001 |
| ***p* for trend** |  | <0.00001 |  | <0.00001 |  | <0.00001 |

Table S6.Weighted characteristics of included vs. excluded adults

|  | Included(n=7642) | Excluded(n=22800) | *P*-value |
| --- | --- | --- | --- |
| Age | 45.83 ± 0.26 | 42.12 ± 0.35 | < 0.001 |
| Gender |  |  | < 0.001 |
| Male | 47.88 | 51.33 |  |
| Female | 52.12 | 48.67 |  |
| Race |  |  | < 0.001 |
| Non-Hispanic White | 67.82 | 62.42 |  |
| Non-Hispanic Black | 10.51 | 12.31 |  |
| Mexican American | 8.42 | 10.91 |  |
| Other Hispanic | 6.11 | 7.18 |  |
| Other race | 7.15 | 7.18 |  |
| Educational level |  |  | 0.002 |
| College Graduate or above | 25.34 | 22.08 |  |
| Less than College Graduate | 74.66 | 77.92 |  |
| Marital status |  |  | 0.010 |
| Married/living with partner | 62.41 | 58.93 |  |
| Widowed/separated | 15.19 | 15.96 |  |
| Never married/divorced | 22.40 | 25.11 |  |
| PIR | 2.91 ± 0.02 | 2.79 ± 0.02 | 0.01 |
| BMI | 28.71 ± 0.09 | 29.27 ± 0.17 | 0.02 |
| Diabetes | 8.47 | 9.89 | 0.04 |
| Smoking |  |  | 0.01 |
| Now | 22.39 | 25.02 |  |
| Some days | 26.32 | 24.91 |  |
| Never | 51.29 | 50.07 |  |
| Urinary iodine concentration（UIC）(ug/mL) | 152 | 141 | 0.051 |
| Frequency of alcohol use in the last 12 months | 68.91 | 66.81 | 0.03 |
| LDL-C | 116.42 ± 0.59 | 117.09 ± 0.70 | 0.08 |
| HDL-C | 50.34 ± 0.29 | 49.91 ± 0.31 | 0.07 |
| Triglycerides | 135.61 ± 1.79 | 138.21 ± 2.02 | 0.09 |

All P-values were calculated under the NHANES complex sampling design using Rao-Scott adjusted x^2^ tests or t-tests.

Table S7. Association between WWI and FT3 after survey-weighted multiple imputation (m = 20)

| Association between WWI and FT3 after survey-weighted multiple imputation (m = 20) | | |
| --- | --- | --- |
| Model | β (95 % CI) | *P*-value |
| Complete case (n = 7 642) | 0.069 (0.045, 0.094) | <0.001 |
| MI (n = 7 642) | 0.068 (0.044–0.093) | <0.001 |

Table S8. Segmented Linear Regression Analysis of WWI and FT3 in Model 2.

| Models | β(95% CI) | *P*-value |
| --- | --- | --- |
| Model 1 |  |  |
| One line effect | 0.054 (0.037, 0.071) | <0.0001 |
| Model 2 |  |  |
| Inflection point（K） | 11.687 |  |
| WWI<=11.687（K） | 0.065 (0.044, 0.086) | <0.0001 |
| WWI>11.687（K） | 0.005 (-0.049, 0.059) | 0.8548 |
| *P* for log-likelihood ratio test |  | 0.064 |

Model I, one-line linear regression; Model II, two-segment regression.

β was the effect size and the 95% CI indicated the confidence interval.

Adjust for:age, gender, race,cycle

Maximum likelihood estimation (MLE) was used under a segmented regression framework. Due to differences in optimization algorithms, there are slight discrepancies compared to the standard survey-weighted linear regression results in Table 2 (<0.01). These differences do not affect the direction of the association or statistical significance (both P<0.001). Segmented regression was used only for exploratory threshold checking. Since P>0.05, the segmented regression results are considered exploratory, and the value 11.687 should not be interpreted as a confirmed biological breakpoint.

Table S9.Segmented Linear Regression Analysis of WWI and FT3 in Model 3.

| Models | β(95% CI) | *P*-value |
| --- | --- | --- |
| Model 1 |  |  |
| One line effect | 0.064 (0.037, 0.092) | <0.0001 |
| Model 3 |  |  |
| Inflection point（K） | 11.687 |  |
| WWI<=11.687（K） | 0.083 (0.049, 0.117) | <0.0001 |
| WWI>11.687（K） | -0.101 (-0.207, 0.005) | 0.0624 |
| *P* for log-likelihood ratio test |  | 0.061 |

Model I, one-line linear regression; Model II, two-segment regression.

β was the effect size and the 95% CI indicated the confidence interval.

Adjust for: age, gender, race, cycle,Education level,marital status,alcohol consumption,diabetes,smoking,UIC,and the PIR.

Maximum likelihood estimation (MLE) was used under a segmented regression framework. Due to differences in optimization algorithms, there are slight discrepancies compared to the standard survey-weighted linear regression results in Table 2 (<0.01). These differences do not affect the direction of the association or statistical significance (both P<0.001). Segmented regression was used only for exploratory threshold checking. Since P>0.05, the segmented regression results are considered exploratory, and the value 11.687 should not be interpreted as a confirmed biological breakpoint.
